# Mutans Streptococci Colonization in Relation to Feeding Practices, Age and the Number of Teeth in 6 to 30-Month-Old Children: An *in vivo* Study

**DOI:** 10.5005/jp-journals-10005-1149

**Published:** 2012-08-08

**Authors:** Rajesh Sharma, AR Prabhakar, Anupama Gaur

**Affiliations:** Associate Professor, Department of Pedodontics and Preventive Dentistry, Jaipur Dental College, Jaipur, Rajasthan, India; Professor and Head, Department of Pedodontics and Preventive Dentistry, Bapuji Dental College and Hospital, Davangere, Karnataka India; Senior Lecturer, Department of Public Health Dentistry, Jaipur Dental College, Jaipur, Rajasthan, India

**Keywords:** Mutans streptococci, Feeding practices, Early childhood caries (ECC), Colony forming units (CFU), Saliva

## Abstract

**Background:** Early childhood caries has been characterized as first affecting the primary maxillary anterior teeth, followed by the involvement of the primary molars. Other terms for dental caries in preschool children, which inappropriately may imply cause for the disease, includes baby bottle tooth decay, nursing caries, milk bottle syndrome, baby bottle caries, nursing bottle mouth and nursing mouth.

**Aim:** To explore the relationships of feeding practices, age and number of teeth present with mutans streptococci colonization in infants.

**Design and setting:** A comparative clinical study conducted on 160 children aged from 6 to 30 months in the Department of Pedodontics and Preventive Dentistry, Bapuji Dental College and Hospital in collaboration with Child Health Institute and Research Center and Department of Oral Pathology and Microbiology, Bapuji Dental College and Hospital, Davangere.

**Materials and methods:** Baseline data collection included: (i) Parents of the infants were asked open ended questions about the baby feeding practices, (ii) The age of the subjects were obtained from the immunization register maintained at Child Health Institute and Research Center and were grouped into group I (6-11 months), group II (12-17 months), group III (18-23 months) and group IV (24-30 months), (iii) Clinical examination of children was done by using mouth mirror and explorer in flash light.^6^ For each child number and location of erupted teeth was recorded, (iv) Microbial screening for mutans streptococci involved sampling of saliva from each child was performed by placing a sterile wooden tongue blade on the dorsum of the tongue and the number of colony forming units (CFU) were recorded.

**Results:** According to feeding practices, 34 children were in breastfed category, 39 were in baby bottle category and 87 children reported no bottle usage. Out of 160 children examined, a total 142 children were colonized with mutans streptococci. 18 children were found to be colonized with low colony forming units, 78 children were found to be colonized with moderate colony forming units and 64 children were colonized with high colony forming units. In baby bottle group, all of 39 subjects were reported to have sweetened milk, sugar in the bottle.

**Conclusion:** Among different feeding practices, all the three subgroups viz breastfed children, children with nursing bottle usage and children with no bottle usage, all have shown mutans streptococci acquisition. But breastfed children have shown least number of high colony forming units, which is increased in the case of children using nursing bottle and is maximum in the children who were neither breastfed nor fed with nursing bottle. Percentage of children colonized with mutans streptococci increases with age and as the number of teeth increase, number of colony forming units were also found to be increasing.

**How to cite this article:** Sharma R, Prabhakar AR, Gaur A. Mutans Streptococci Colonization in Relation to Feeding Practices, Age and the Number of Teeth in 6 to 30-Month-Old Children: An *in vivo* Study. Int J Clin Pediatr Dent 2012;5(2): 124-131.

## INTRODUCTION

Early childhood caries (ECC) is a term that describes dental caries in infants and toddlers, and is attributed, in part, to prolonged and inappropriate feeding with a baby bottle. The condition also has been associated with breastfeeding, sweetened pacifier use and frequent use of sweetened medicaments.^1^

Early childhood caries has been characterized as first affecting the primary maxillary anterior teeth, followed by the involvement of the primary molars. Other terms for dental caries in preschool children, which inappropriately may imply cause for the disease, includes baby bottle tooth decay, nursing caries, milk bottle syndrome, baby bottle caries, nursing bottle mouth and nursing mouth.^2,3^

There is a general agreement that the oral cavity of man is sterile at birth, but is soon contaminated with a flora predominantly streptococcal in nature.^4,5^ Mutans streptococci is strongly associated with carious lesions in man and laboratory animals and have been implicated in the initiation of carious lesions, while lactobacilli are believed to be involved in its progression. They are considered to be one of the prime etiologic agents of dental caries.^4,6-9^ Mutans streptococci require the presence of hard, nondesquamating surface for their colonization. Therefore, predentate infants do not harbor this organism.^4,6,10,11^

Saliva is the vehicle by which the transfer occurs. A mother with high number of mutans streptococci in her saliva is a source for infection in the close vicinity of the child. If she, for example, uses her own spoon to feed the child, she may introduce in time, several hundreds of colony forming units into the mouth of the child.^3,12^

Microbiological studies of nursing caries in bottle-fed children have demonstrated heavy colonization of the affected teeth by mutans streptococci and lactobacilli.^13^ Instances of nursing caries associated with breastfeeding have been reported in studies by Brams and Maloney (1983) and Heckett et al (1984).^13^

The recent development of a reliable selective medium for *Streptococcus mutans* has permitted a more precise determination of the time of appearance of *Streptococcus mutans* in relation to the development of the primary dentition. Using this more sensitive technique it was possible to demonstrate that *Streptococcus mutans* can be detected in infants soon after eruption of the primary incisors, but not in normal predentate infants.^4^

**Graph 1:**
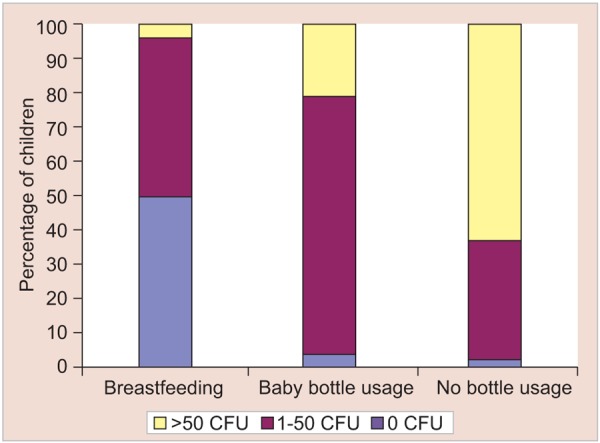
Percentage of children with mutans streptococci colonization by feeding practices

Children with early childhood caries may have a history of sleeping with a bottle or using a bottle or breast beyond the normal weaning time, yet there is also evidence that some children who sleep with a bottle do not develop early childhood caries. Such feeding practices may vary depending on the age of the child, but most studies do not differentiate feeding practices at various ages (Graph 1). Other factors also may be involved in disease initiation, since some children do not manifest caries on their maxillary anterior teeth until 4 to 5 years of age, after which time bottle use generally has been discontinued.^2^

**Graph 2:**
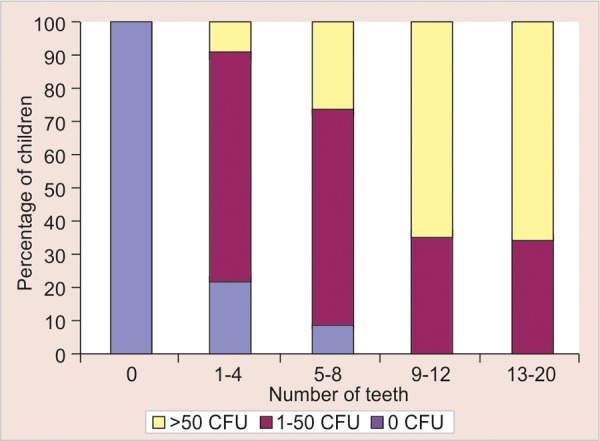
Percentage of children with mutans streptococci colonization by number of teeth

An understanding of the relationship between feeding practices and age with the acquisition of mutans streptococci is necessary to better comprehend the mechanisms of early childhood caries, and consequently to prevent it. The purpose of this study therefore was to explore the relationships of feeding practices, age and number of teeth present with mutans streptococci colonization in infants (Graph 2).

## MATERIALS AND METHODS

### Study Design

A cross-sectional analytical study, conducted on 160 children aged from 6 to 30 months which were randomly selected with no significant medical problem, who were accompanied by their parents visiting Outpatient Department of Child Health Institute and Research Center, Davangere, for the purpose of immunization.

### Ethical Clearance

Ethical clearance was taken from the Ethical Committee of Bapuji Dental College, Davangere and voluntary informed consent was obtained from the parents of the children.

### Materials

**Fig. 1:**
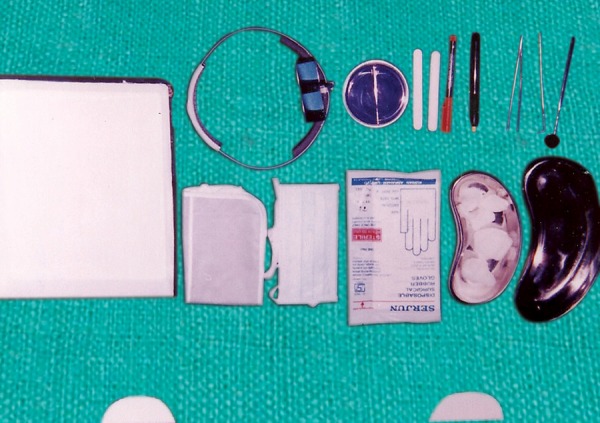
Armamentarium

**Fig. 2:**
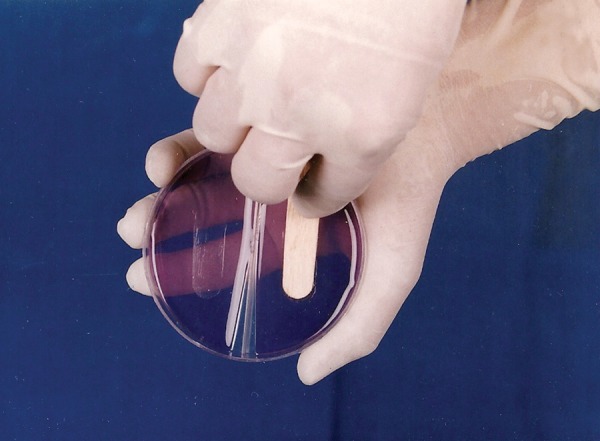
Sterile wooden tongue blades

**Fig. 3:**
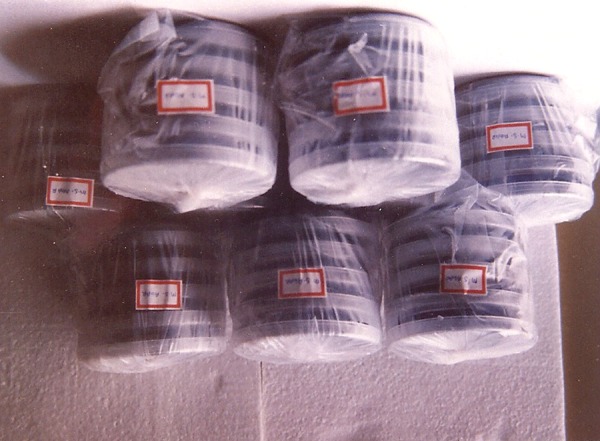
Mitis salivarius agar plates

**Fig. 4:**
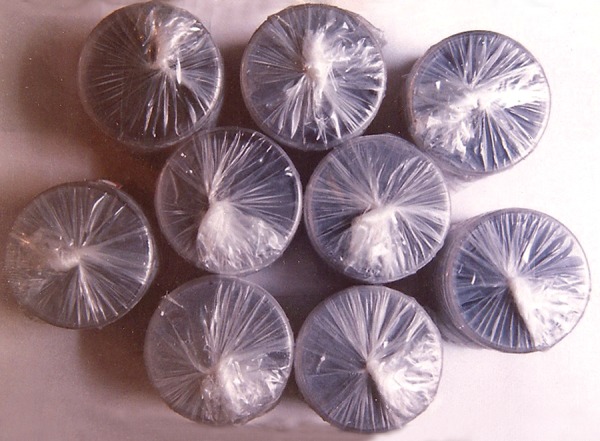
Mitis salivarius agar plates (above view)

- Armamentarium (Fig. 1)
- Sterile wooden tongue blades (Fig. 2)
- Mitis Salivarius agar plates (Delta Biological Ltd., Bengaluru) (Figs 3 and 4).

### Equipments

**Fig. 5:**
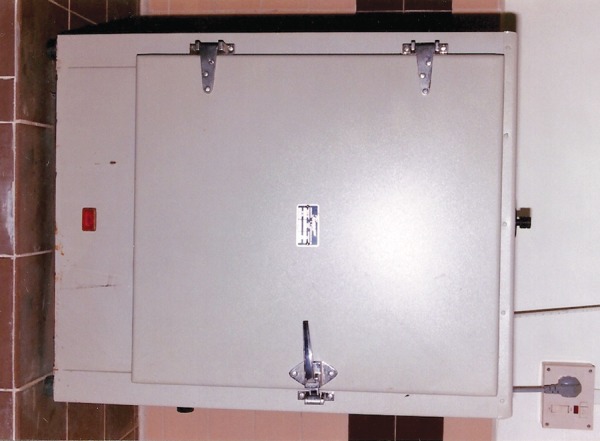
Incubator (Tempo)

**Fig. 6:**
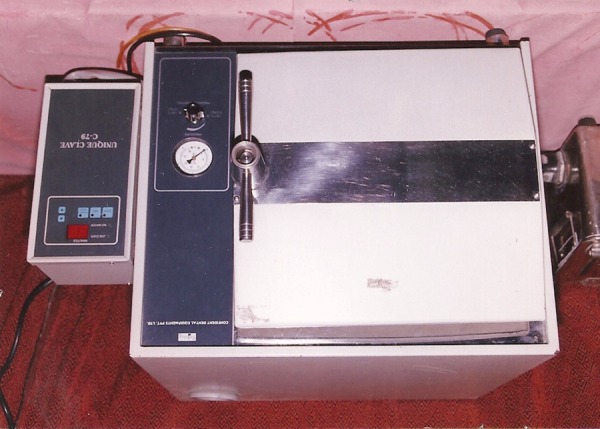
Autoclave (Unique Clave C-79)

- Incubator (Tempo) (Fig. 5).
- Autoclave (Unique Clave C-79) (Fig. 6).

## STUDY PROCEDURE

**Fig. 7:**
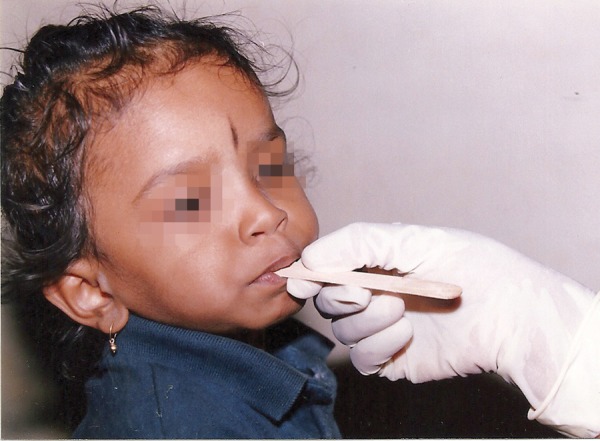
Microbial screening for mutans streptococci

- Parents of the infants were asked open ended questions about the baby feeding practices which are as follows:
  1. Whether the child is on breastfed ?
  2. Whether the child is fed with baby bottle ? If fed with a baby bottle, what food items were put in bottle during the last week.
    - Milk/milk formula
    - Sweetened beverages
  3. No bottle – that is the child reportedly was neither bottle fed nor breastfed in the last week but resorted to other means of feeding like spoonfeeding, tumbler feeding.
- The age of the subjects were obtained from the immunization register maintained at Child Health Institute and Research Center and were grouped as follows:
  *Group I:* 6 to 11 months
  *Group II:* 12 to 17 months
  *Group III:* 18 to 23 months
  *Group IV:* 24 to 30 months
- Clinical examination of children was done by using mouth mirror and explorer in flash light.^6^
  For each child number and location of erupted teeth was recorded. In this study, a tooth was considered present if cusp tips and central fossa were exposed to the oral cavity. The presence or absence of caries was also noted.
- *Microbial screening for mutans streptococci:* Microbial screening for mutans streptococci involved sampling of saliva from each child by placing a sterile wooden tongue blade on the dorsum of the tongue until the tongue blade was visibly moistened (Fig. 7). The tongue blade was then impressed on to the plate containing the selective media for isolation of mutans streptococci - Mitis Salivarius agar. Each agar plate was numbered by using permanent writing pen. These plates were incubated anaerobically at 37°C for 72 hours, after which time bacterial growth was assessed by visually counting the number of colony forming units (CFU) resembling mutans streptococci that appeared within the impression.^1^

The number of colony forming units (CFU) were recorded as:

**Table:** 

*Groups*		*Colony forming units*		*Photograph*	
Low		0 CFU		Figure 8	
Moderate		1-50 CFU		Figure 9	
High		>50		Figure 10	

### Statistical Analysis

Chi-square test was used to analyze the categorical data. A p-value of less than 0.05 was considered statistically significant.

## RESULTS

Table 1 shows the distribution of children according to age, number of teeth present and feeding practices. Fourty children were taken in each age group. According to number of teeth presents nine children were with 0 number of teeth and 23, 54, 14 and 60 number of children were with 1 to 4, 5 to 8, 9 to 12 and 13 to 20 number of teeth respectively. According to feeding practices, 34 children were in breastfed category, 39 were in baby bottle category and 87 children reported no bottle usage.

**Fig. 8:**
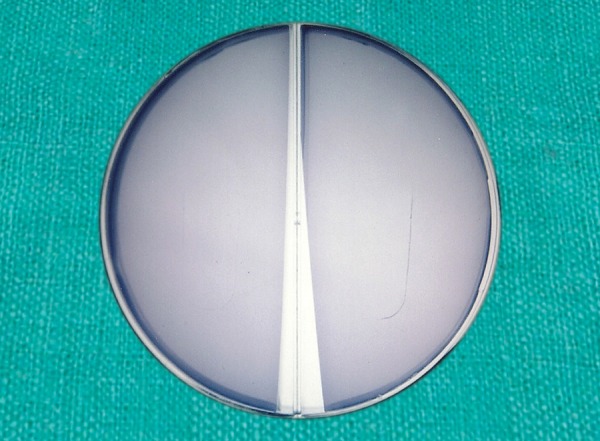
Tongue blade impressed on agar plates

**Fig. 9:**
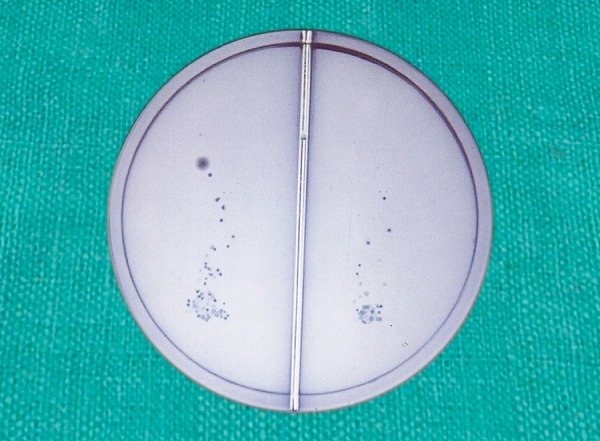
Low colony forming units for mutans streptococci

**Fig. 10:**
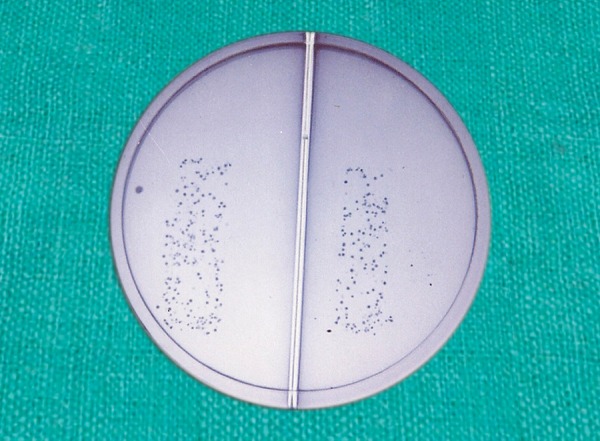
High colony forming units for mutans streptococci

**Graph 3:**
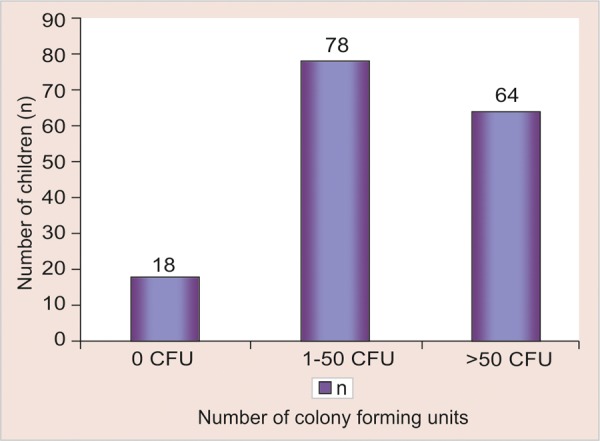
Total percentage of children colonized with mutans streptococci

Table 2 shows the distribution of the children with respect to mutans streptococci colonization. Out of 160 children examined, a total 142 children were colonized with mutans streptococci (Graph 3). Eighteen children were found to be colonized with low colony forming units, 78 children were found to be colonized with moderate colony forming units and 64 children were colonized with high colony forming units.

Table 3 shows the distribution of children with mutans streptococci by feeding practices. It demonstrates that 34 children were reported to be on exclusively breastfed, in which 16 (47.0%), 17 (50%) and one (3.0%) were with low, moderate and high colony forming units respectively. Around 39 children were reported to be baby bottle fed, in which one (2.6%), 30 (76.9%) and eight (20.5%) were with low, moderate and high colony forming units respectively and 87 children were reported to be on no bottle usage, out of which one (1.1%), 31 (35.6%) and 55 (63.2%) were with low, moderate and high colony forming units respectively.

The table also demonstrate the least number of high colony forming units in the children exclusively on breastfeeding which has increased in case of baby bottle usage and maximum in case of children on no bottle usage. Statistically, the results were found to be highly significant (p < 0.001). In baby bottle group, all of 39 subjects were reported to have sweetened milk sugar in the bottle.

**Graph 4:**
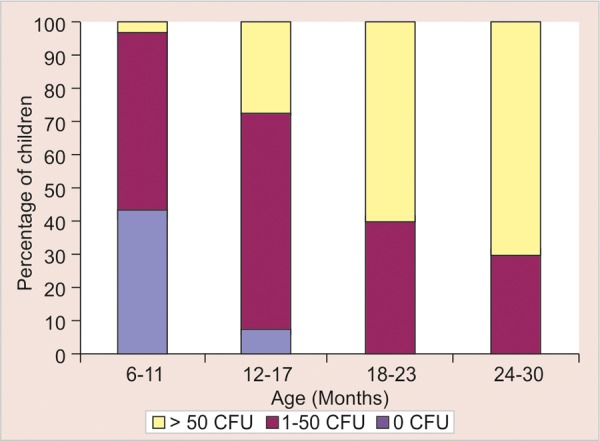
Percentage of children with mutans streptococci colonization by age categories

Table 4 shows the distribution of child with mutans streptococci by age categories (Graph 4). In 6 to 11 months age group children, 16 (40%), 23 (57.5%) and one (2.5%) were found with low, moderate and high colony forming units respectively. In 12 to 17 months age group, two (5%), 27(67.5%) and 11 (27.5%) were found to be with low, moderate and high colony forming units respectively. In 18 to 23 months age group, 16 (40%) and 24 (60%) were found to be with moderate and high colony forming units respectively. In 24 to 30 months age group, 12 (30%) and 28(70%) were found to be with moderate and high colony forming units. The results were statistically significant (p < 0.001). The figures demonstrate that as the age advances from 6 months toward 30 months, the number of children in the high level of mutans streptococci category were found to be increasing.

**Table 1:** **Table 1:** Number of children by each categorization variable

*Age (months)*		*N*	
6-11		40	
12-17		40	
18-23		40	
24-30		40	
Total		160	
*Number of teeth*		*N*	
0		9	
1-4		23	
5-8		54	
9-12		14	
13-20		60	
Total		160	
*Feeding practices*		*N*	
Breastfeeding (BF)		34	
Baby bottle (BB)		39	
No bottle (NB)		87	
Total		160	

**Table 2:** **Table 2:** Number of children affected by mutans streptococci colonization

*Colony forming units (CFU)*		*N*	
0 CFU (low)		18	
1-50 CFU (moderate)		78	
> 50 CFU (high)		64	
Total		160	

Table 5 demonstrates the distribution of children with the mutans streptococci colonization by number of teeth. Children with 0 number of teeth did not show mutans streptococci colonization. Children with 1 to 4 number of teeth have shown five (21.7%), 16 (69.6%) and two (8.7%) with low, moderate and high colony forming units respectively. Children with 5 to 8 number of teeth have shown four (7.4%), 36 (66.7%) and 14 (25.9%) with low, moderate and high colony forming units respectively. Children with 9 to 12 number of teeth have shown five (35.7%) and nine (64.3%) with moderate and high colony forming units respectively. Children with 13 to 20 number of teeth have shown 21 (35.0%) and 39 (65.0%) with moderate and high colony forming units respectively. The observations demonstrate that as the number of teeth increased from 0 to 20, the number of children with high level of mutans streptococci were found to be increasing. These observations were statistically highly significant with p-value of p < 0.001.

## DISCUSSION

Caries in early childhood has been a challenge to the dental profession throughout the developing and developed world. Although there have been major advancements in the fields of pathogenesis and prevention of dental caries in the last two decades, still there are reports of a high prevalence of caries in preschool children. This means there is a serious problem of caries in early childhood, i.e. infants and young children.^14^

**Table 3:** **Table 3:** Distribution of children with mutans streptococci by feeding practices

*Feeding practices*		*0 CFU (low)*		*1-50 CFU (moderate)*		*>50 CFU (high)*		*Total*	
Breastfeeding (BF)		16 (47.0%)		17 (50.0%)		1 (3.0%)		34	
Baby bottle (BB)		1 (2.6%)		30 (76.9%)		8 (20.5%)		39	
No bottle (NB)		1 (1.1%)		31 (35.6%)		55 (63.2%)		87	
Total		18		78		64		160	

χ^2^ = 85.8, p < 0.001, Highly significant

**Table 4:** **Table 4:** Distribution of children with mutans streptococci by age categories

*Age (months)*		*0 CFU (low)*		*1-50 CFU (moderate)*		*>50 CFU (high)*		*Total*	
6-11		16 (40.0%)		23 (57.5%)		1 (2.5%)		40	
12-17		2 (5%)		27 (67.5%)		11 (27.5%)		40	
18-23		0.0 (0%)		16 (40%)		24 (60%)		40	
24-30		0.0 (0%)		12 (30%)		28 (70%)		40	
Total		18		78		64		160	

χ^2^ = 75.4, p < 0.001, Highly significant

**Table 5:** **Table 5:** Distribution of children with mutans streptococci by number of teeth

*Number of teeth present*		*0 CFU (low)*		*1-50 CFU (moderate)*		*>50 CFU (high)*		*Total*	
0		9 (100.0%)		0.0 (0%)		0.0 (0%)		9	
1-4		5 (21.7%)		16 (69.6%)		2 (8.7%)		23	
5-8		4 (7.4%)		36 (66.7%)		14 (25.9%)		54	
9-12		0.0 (0%)		5 (35.7%)		9 (64.3%)		14	
13-20		0.0 (0%)		21 (35.0%)		39 (65.0%)		60	
Total		18		78		64		160	

χ^2^ = 110.4, p < 0.001, Highly significant

The notion that dental caries in animals is an infectious, transmissible disease was first demonstrated by Keyes (1960). Since, that time group of phenotypically similar bacteria, collectively known as the mutans streptococci (MS), has been implicated as the principal bacterial component responsible for dental caries in humans.^7^ Infants do not harbor this organism until sometime after teeth emerge.^10^ Mutans streptococci require the presence of a hard, nondesquamating surface for their colonization.^6^

Although, it is logical to assume that the infectious and transmissible characteristics of dental caries in animal models applies to the disease in human beings, there is little direct evidence to support this concept. Notably lacking are data concerning acquisition and transmission of mutans streptococci.^4^

It was believed for long that the oral cavity was sterile at birth.^5,11^ However, it is logical to comprehend that acquisition of mutans streptococci by the infants must have some dependence on the factors like age, number of teeth present and feeding practices, which have been shown in various studies.^1,4,6,7,10,11,15-17^

The association of mutans streptococci in the initiation of early childhood caries has been proved beyond doubt. An understanding of the relationship between feeding practices, age and number of teeth present with the acquisition of mutans streptococci is necessary to better comprehend the mechanisms of early childhood caries and consequently to prevent it. The purpose of this study therefore was to explore the relationships of feeding practices, age and number of teeth present with mutans streptococci colonization in infants.

In the present study the subjects who were between 6 to 30 months were selected. This period was found to be ideal to establish the relationship between three study parameters as 6 months marks the initiation of tooth eruption and the assisted feeding would be still present up to 30 months of age.

The Mitis Salivarius agar medium was choosen in this study to cultivate the microorganism as this medium is specific for the growth of mutans streptococci.^6,8,18,19^

Children included in this investigation were from Davangere district, therefore the findings may not be generalizable to all the children. Information about the feeding practices is based on parental self-reports using open ended questions and, as with any self-reported data may result in some level of exposure misclassification.

One of the finding of the present study was that, among different feeding practices, all the three subgroups viz breast fed children, children with nursing bottle usage and children with no bottle usage, all have shown mutans streptococci acquisition. But breastfed children have shown least number of high colony forming units, which is increased in the case of children using nursing bottle and is maximum in the children who were neither breastfed nor fed with nursing bottle. This observation can be explained on the basis that in the bottle fed and no bottle group, the feeds were mixed with high degree of sugar. This finding of increased mutans streptococci count in children consuming sugar with milk is similar to that made by Kreulen CM et al (1997).^20^ The observation from the present study that the percentage of children colonized with mutans streptococci increases with age, can be explained on the basis that as the age advances, there is a change in feeding practices that is breastfeeding is seen in early months which is followed by nursing bottle feeding and there after by no bottle usage. The later two feeding groups have shown high prevalence of mutans streptococci acquisition. This observation is similar to that of Kreulen CM et al (1997)^20^ and Houte JV et al (1982).^7^

Another observation from the present study was that as the number of teeth increase, number of colony forming units were also found to be increasing. This can be due to the fact that with the advancement of age, the number of teeth are bound to increase hence providing more nonshedding surface for the colonization of the microorganisms. This is in agreement with the findings observed by Berkowitz et al (1976),^4^ and Brown et al (1985).^21^

The present study was conducted in the Bapuji Dental College and Hospital, in collaboration with Child Health Institute and Research Center, and Department of Oral Pathology and Microbiology, Bapuji Dental College and Hospital, Davangere.

The aim of the present study was to know the relationship between feeding practices, age and number of teeth present with colonization of mutans streptococci in 6 to 30 months old children.

Parents were asked questions about baby feeding practices which included breastfeeding, usage of baby bottle and no bottle usage. Children were examined by using mouth mirror and explorer in flashlight. For each child, number and location of teeth was noted and caries was recorded for presence or absence. Microbial screening for mutans streptococci involved sampling saliva from each child by placing a sterile wooden tongue blade on the dorsum of the tongue until the tongue blade was visibly moistened. The tongue blade was then impressed on to the plate containing selective media for mutans streptococci – mitis salivarius agar. These plates were incubated anaerobically at 37°C for 72 hours, after which time bacterial growth was assessed by visually counting the number of colony forming units (CFU) resembling mutans streptococci that appeared within the impression. The number of colony forming units (CFU) were recorded as:

      Low: 0 CFU

      Moderate: 1 to 50 CFU

      High: > 50 CFU

## CONCLUSION

Following conclusions were drawn from this study:

1. Children on no bottle usage have shown the highest acquisition of mutans streptococci colonization, which was less in the case of baby bottle usage and least in breastfed children.
2. With increase in the age of children, the percentage of children with mutans streptococci colonization has increased.
3. Mutans streptococci colonization increased with the increase in the number of teeth.

However, additional investigations are needed to verify and further explore the identified relationships.
